# Tertiary lymphoid organs in the inflammatory myopathy associated with PD-1 inhibitors

**DOI:** 10.1186/s40425-019-0736-4

**Published:** 2019-09-18

**Authors:** Shiro Matsubara, Morinobu Seki, Shigeaki Suzuki, Takashi Komori, Mikio Takamori

**Affiliations:** 1 Department of Neurology, Tokyo Metropolitan Neurological Hospital, 2-6-1 Musashidai, Fuchu, Tokyo, 183-0042 Japan; 2 Department of Neurology, Keio University School of Medicine, 35 Shinanomachi, Shinjuku-ku, Tokyo, 160-8582 Japan; 3 Department of Neuropathology, Tokyo Metropolitan Neurological Hospital, 2-6-1 Musashidai, Fuchu, Tokyo, 183-0042 Japan; 4 Respiratory / Medical Oncology Department, Tokyo Metropolitan Tama Medical Center, 2-8-29 Musashidai, Fuchu, Tokyo, 183-0042 Japan

**Keywords:** PD-1 inhibitor, Adverse effect, Inflammatory myopathy, Tertiary lymphoid organ, Cytotoxic T cell, High endothelial venule

## Abstract

**Background:**

Programmed cell death 1 inhibitors have revolutionized therapy for cancer by their outstanding effectiveness. However, they may cause adverse effects, among which inflammatory myopathy is one of the most disabling. To elucidate its mechanism, we analysed muscle biopsies and compared them with other inflammatory myopathies.

**Methods:**

Muscle biopsies from three patients with inflammatory myopathy after treatment with PD-1 inhibitors for cancer were subjected to immunohistochemical and ultrastructural analyses to localize CD8+ cytotoxic cells and markers of lymphoid follicles. For comparison, two cases of polymyositis and one of juvenile dermatomyositis were examined.

**Results:**

Nearly identical pathological features were observed in the three cases. In the island-like foci of inflammation, muscle fibers were undergoing degeneration. CD8+ cytotoxic T cells, macrophages, CD4+ cells, and B cells were observed in the foci. CD8+ cells were seen outside and inside the basal lamina of non-necrotic muscle fibers. Lymphoid follicle-like structures with CD21+ follicular dendritic cells were present. The blood vessels in the foci showed features consistent with the high endothelial venules, on which their markers, PNAd and CCL21, were expressed. In polymyositis, blood vessels stained only faintly for PNAd and CCL21, while in juvenile dermatomyositis, in which tertiary lymphoid follicle-like structure was reported in the past, they stained positively.

**Conclusions:**

In inflammatory myopathy associated with PD-1 inhibitors, CD8+ cells appear to predominantly destruct muscle fibers. The presence of lymphoid follicle-like structures and expression of PNAd and CCL21 on the endothelial cells suggest the tertiary lymphoid organs are formed, and involved in the leakage of lymphocytes. Thus, in the three cases examined, formation of the tertiary lymphoid organs is likely to play an important role in genesis of the PD-1 myopathy.

## Introduction

Blockade of tumor immune evasion with programmed cell death 1(PD-1) inhibitors has yielded significant success in therapy for melanoma and a wide variety of other tumors [1]. However, among its adverse effects, inflammatory myopathy [2, 3] is one of the most disabling.

Cytotoxic T cells and natural killer cells play pivotal roles in the immune reaction against tumor. In the tumor tissue, CD8+ cells migrate from the blood vessel to the tissue through the vessel wall. This process of vascular leakage is an important step in tumor immunity and takes place at special sites of blood vessel called the lymph node-like vasculature or tertiary lymphoid organ (TLO) [4]. In the peripheral lymph nodes, which are the secondary lymphoid organs, vascular leak occurs at high endothelial venules (HEVs), where peripheral node addressin (PNAd) and chemokine ligand 21 (CCL21) are expressed on the endothelial cells. In a mouse model of malignant tumor tissue, activated naïve T cells can not only induce lymph node-like vasculature and leak into the tumor tissue, but can also destroy tumor tissue [5].

PNAd is a glycoprotein with the MECA-79 epitope and a ligand for L-selectin. CCL21 and CCL19 are ligands of chemokine receptor CCR7 which is expressed on the surface of activated lymphocyte and is involved in lymph node homing of naïve and regulatory T cells via HEVs in the lymph node [6]. CCL21 is chemotactic for activated T cells.

Island-like scattered foci of inflammation and degeneration of muscle fibers, seemingly a hallmark of myopathy associated with PD-1 inhibitor (PD-1 myopathy) [3], might reflect a unique mechanism of the condition. We examined the possible involvement of vascular leakage of lymphocytes from the blood vessels because it is known to occur in tumor tissues.

## Patients and methods

### Patients

Muscle biopsies from three patients were examined. In addition to routine histological studies, histochemical, immuno-histological examinations, and ultrastructural studies, partly applying immuno-electron microscopic studies, were performed. For comparison, biopsies from cases of polymyositis (PM) and juvenile dermatomyositis (JDM) were examined.

#### Case 1

A 57- year-old male with adenocarcinoma of the lung was treated with 2 cycles of nivolumab 3 mg/kg. His serum creatine kinase activity (CK) was found to be raised to 2637 IU/L (normal < 200 IU/L) 19 days later. Needle electromyography (EMG) showed myopathic changes. A moderate weakness of neck flexor muscles and proximal muscles of the limbs was present. Muscle biopsy from the biceps brachii muscle was examined.

#### Case 2

A 63- year-old female was treated for head and neck carcinoma with three cycles of nivolumab 3 mg/kg. Thirty days later, she developed dropped head, dysarthria and weakness in proximal muscles with myalgia. Her CK was 3021 IU/L. EMG showed myopathic changes while MRI of the skeletal muscles revealed high intensity areas on the T2-weighted images. Muscle biopsy was taken from the triceps brachii muscle.

#### Case 3

A-73-year-old male was treated for lung adenocarcinoma with two cycles of pembrolizumab 200 mg. Twenty-five days later, he developed muscle weakness of the lower limbs. His CK was 1643 IU/L. EMG showed myopathic changes while muscle MRI was normal. A muscle biopsy was taken from the rectus femoris muscle.

Muscle biopsies from two patients with PM, and one patient with JDM were studied for localization of PNAd and CCL21. Diagnosis of PM was made according to the criteria of Dalakas and Hohlfeld [7]. JDM was diagnosed based on criteria of the EULAR / ACR [8]. These three patients showed inflammatory changes in their muscles comparable to those of the present cases of PD-1 myopathy. Clinical and histological features of the three control cases are presented in the Additional file 1. None of patients examined in this study was treated with corticosteroids or other immune-modulating agents before muscle biopsy except PD-1 inhibitors.

Written informed consent to the muscle biopsy was obtained from patients or a parent for the sake of diagnosis, along with their consent to the use of the specimen for research purposes. This research was approved by the Medical Ethic Committee of Tokyo Metropolitan Neurological Hospital.

## Methods

### Histological examination

Routine histological examination and transmission electron microscopy for inflammatory myopathy were performed as described previously [9].

### Immunofluorescence studies

Localization of CD21+ cells: Formalin-fixed paraffin sections were subjected to the wet heat-induced epitope retrieval [10]. Anti-CD21 rabbit monoclonal antibody (Arigo) was applied followed by anti-rabbit IgG goat polyclonal antibody (GeneTex) labeled with FITC.

Localization of CD8+ cells: All following immunofluorescence studies were performed on the frozen sections. A mixture of anti-human CD8 mouse monoclonal antibody (Dako) and anti-laminin rat monoclonal antibody (ICN) was applied. Then a mixture of anti-mouse IgG goat polyclonal antibody labeled with rhodamine (GeneTex) and anti-rat IgG goat polyclonal antibody labeled with FITC (Novus) was applied.

Localization of PNAd: Anti-PNAd rat monoclonal antibody (MECA79) (Novus) was applied along with control sections on which normal rat serum was applied. After washing, anti-CD31 mouse monoclonal antibody (Proteintech) was applied. Then a mixture of anti-rat IgM goat polyclonal antibody labeled with FITC (Novus) and anti-mouse IgG goat polyclonal antibody labeled with rhodamine (GeneTex) were applied.

Localization of CCL21: Anti-CCL21 rabbit polyclonal antibody (Bioworld) was applied along with control sections on which normal rabbit serum was applied. After washing, anti-CD31 mouse monoclonal antibody (Proteintech) was applied. After washing, a mixture of anti-rabbit IgG goat polyclonal antibody labeled with FITC and anti-mouse IgG goat polyclonal antibody labeled with rhodamine (GeneTex) were applied.

### Immuno-electron microscopic study to localize CD8+ cells

Frozen sections, 8 μm thick, were cut from muscle biopsies from three cases of PD-1 myopathy and one case without pathological change. A pre-embedded immunoelectron microscopic study [11] was carried out with minor modifications. Details of method are described in the Additional file 2.

## Results

### Light microscopy and immunohistochemistry

**Fig. 1:**
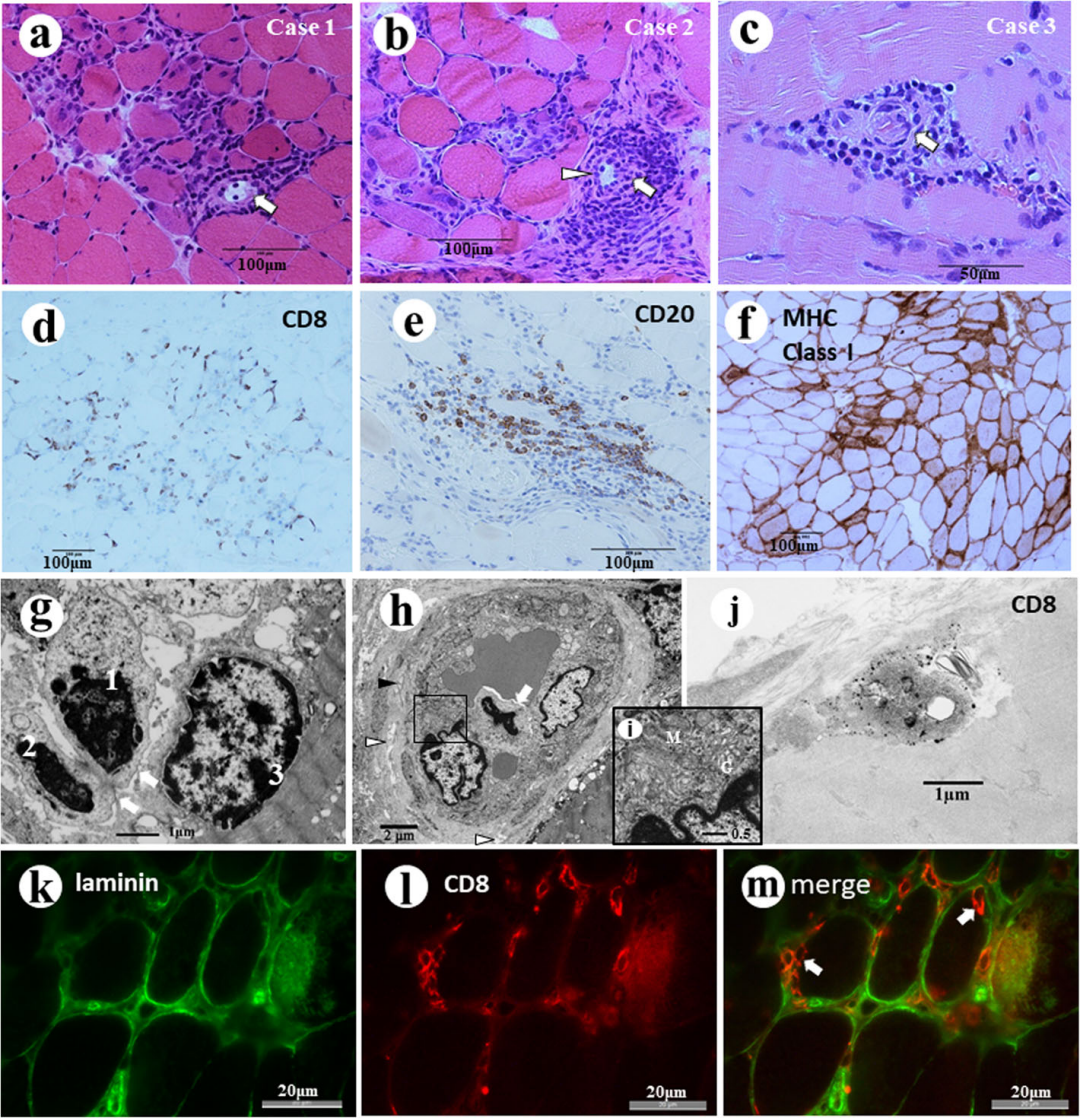
**a**, **b**, **c** Foci of inflammatory cell infiltration in three cases of PD-1 myopathy. In the inflammatory foci, lymphoid follicle-like structures with area like germinal center (arrow). Some blood vessels in the lymphoid follicle-like structures have endothelial cells with ample cytoplasm simulating high endothelial venules (arrowhead). **d** CD8 + cells were seen in the endomysium. Many of them were in close contact with the muscle fiber surface (case 2). **e** CD20+ B lymphocytes are seen in the foci of inflammation in all three cases (case 2). **f** MHC class I antigen is expressed on the surface of almost all muscle fibers (Case 1). **g** Electron micrograph of Case 2. In the foci of inflammation, two mononuclear cells (1,2) are located under the basal lamina of a muscle fiber and in contact with the plasma membrane (arrows) of a muscle fiber (3). **h** In the Case 2, the transverse view of a blood vessel located in a focus of inflammation shows a mononuclear cell (arrow) in direct contact with two endothelial cells protruding its tip into the vessel wall. The vessel has a basal lamina, which is irregular in thickness and rough in texture with many spaces (white arrowheads) and occasional fibrous structures (black arrowhead) in its matrix. **i** Higher magnification of the highlighted area in Fig. 1e. The endothelial cells have ample cytoplasm containing abundant Golgi complexes (G), mitochondria (M) and ribosomes. **j** Immuno-electron micrograph of Case 2. A cell underneath the cell surface of a muscle fiber has numerous electron dense particle of size consistent with diameters of gold particles labeled to the anti-CD8 antibody. **k**-**m** Immunofluorescence study of Case 2. Anti-laminin antibody labeled with FITC (green) shows the basal lamina of muscle fibers and blood vessels. The merged image shows CD8+ cells (orange) in and around muscle fibers, including some located inside the basal lamina of the muscle fibers (arrows)

Muscle biopsies from three cases showed nearly identical pathological features with scattered island –like foci of inflammation and degeneration and regeneration of muscle fibers as reported previously [3]. Furthermore, a small number of lymphoid follicle-like structures, some accompanying HEV-like blood vessels were seen in all cases (Fig. 1a-c). One each lymphoid follicle-like structures in cases 1 and 2, and two follicle-like structures were seen in case 3. When calculated from width of sections, one lymphoid follicle-like structure was seen in 13.5 square millimeters of the sections in average. Perifascicular atrophy and rimmed vacuoles were not seen. The infiltrating cells are composed of similar numbers of CD8+ T cells (Fig. 1d), CD4+ T cells, CD68+ macrophages, and CD20+ B cells (Fig. 1e). Aberrant expression of the major histocompatibility complex class I antigen was observed at the surface of almost all muscle fibers, but it was particularly strong on the fibers in and around the foci of inflammation (Fig. 1f).

### Transmission electron microscopy

Muscle fibers showed a wide variety of degenerative changes including streaming of z lines, loss of myofilaments and necrosis. Myonuclei also showed degeneration. Neuromuscular junctions observed in Case 1 showed no definite abnormality (not illustrated). At foci of inflammatory cell infiltration, mononuclear cells were mainly seen in the interstitial tissue and did not show any tendency to fuse to each other. Some of the mononuclear inflammatory cells were seen inside of the basal lamina of the muscle fibers which were either undergoing degeneration or appeared nearly normal. Some of them were in direct contact with the sarcoplasm of the muscle fiber (Fig. 1g). The blood vessels in the foci of inflammation (Fig. 1h) often had ample cytoplasm which contained prominent Golgi complexes and mitochondria (Fig. 1i). The basal lamina around the vessel were irregular in thickness and had a coarse texture with many spaces and fibrous structures. In the luminal surface of the endothelial cells, mononuclear cells were sometimes seen in direct contact with the endothelial cells (Fig. 1h).

### Immuno-electron microscopy

Diameters of the gold particles labeled to the antibody were measured under the electron microscope beforehand. Many of the particles were 25 to 30 nm in diameter, but larger particles 50 to 60 nm were also seen (not illustrated). The latter particles were estimated to be aggregates produced in the process of tissue preparation [11].

Nearly identical findings were observed in muscle biopsies from three cases of PD-1 myopathy. Some of the mononuclear cells under the basal lamina of the muscle fibers showed on their surface positive deposition of gold particles 30 to 60 nm in diameter, consistent with those conjugated with anti-CD8 antibody (Fig. 1j). The control muscle with no pathological change did not show any specific deposition of the gold particles.

### Immunofluorescence microscopy

Localization of CD8+ cells: Many of the infiltrating cells at the foci of inflammation and muscle degeneration were positive for CD8. Some of the positive cells were located inside the basal lamina of the muscle fibers, which was demonstrated by the anti-laminin antibody (Fig. 1k-m).

**Fig. 2:**
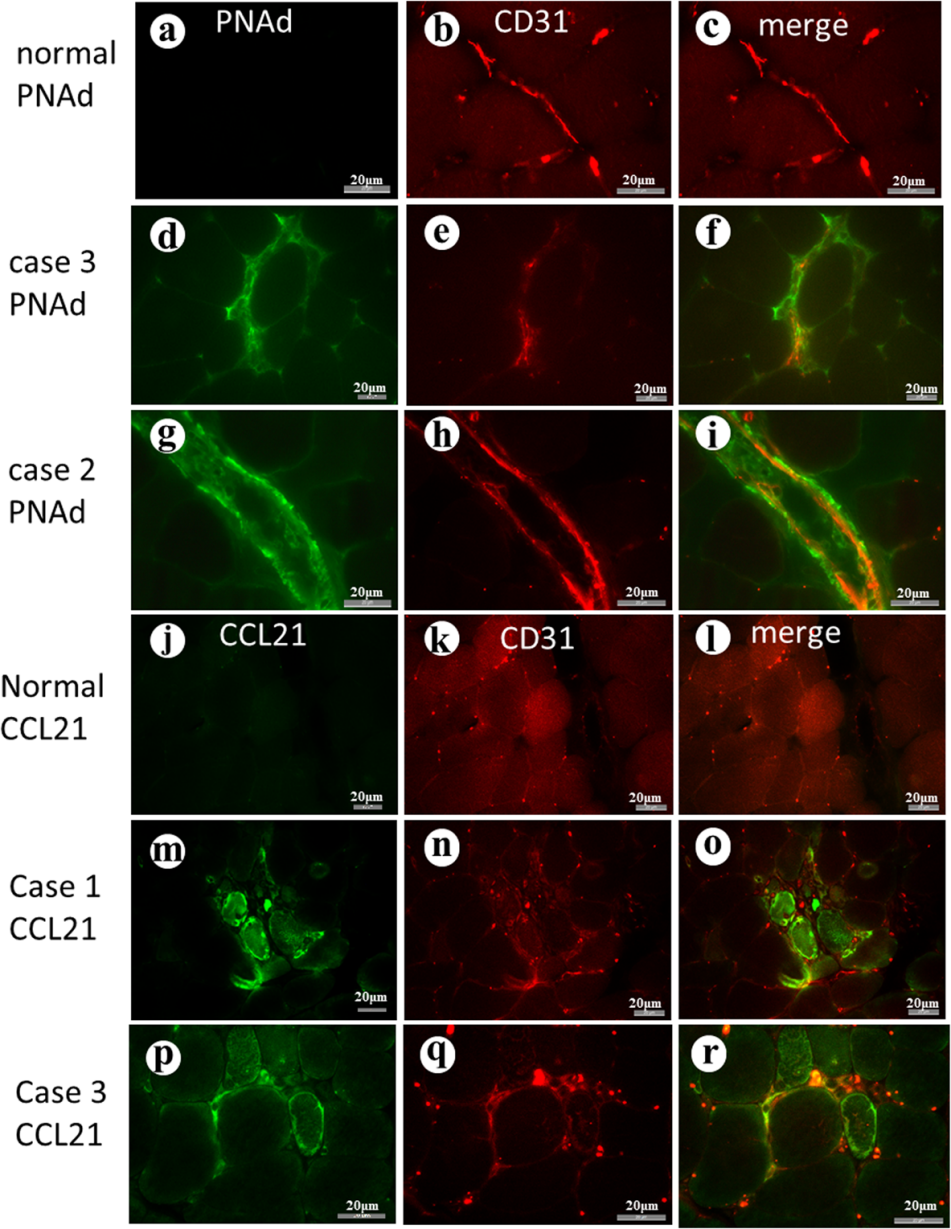
**a**-**c** PNAd and CD31(endothelial cells) in a control muscle without pathological change. PNAd is not visible. **d**-**f** PNAd in Case 3 showing positivity (green) in the interstitial tissue, particularly on the blood vessels (red). **g**-**i** PNAd in Case 2. The merged image demonstrates a vessel strongly positive for PNAd (green) on the endothelial cells (red). **j**-**l** CCL2 in normal muscle. No positivity was detected. **m**-**o** CCL21in Case 1. The merged image shows positive CCL21 (green) on some blood vessels (red) in the perivascular space and around the muscle fibers. **p**-**r** CCL21 in Case 3. Blood vessels (CD31 red) around a muscle fiber show positivity for CCL21

Localization of PNAd and CCL21: In control muscle without pathological change, no positivity for PNAd (Fig. 2 a-c) or CCL21 (Fig. 2j-i) was detected. No specific positivity was seen on the control sections on which normal rat serum or normal rabbit serum was applied respectively instead of primary antibodies (Additional file 1: Figure S2). In three cases of PD-1 myopathy, many of the blood vessels in the foci of inflammation showed positivity for PNAd. The positivity was observed at the vascular wall, perivascular space or both, and it varied greatly in length along the vessels (Fig. 2d-i). Positivity for CCL21 was seen in cases of PD-1 myopathy at the endothelial cells of the blood vessels, but also in the perivascular space and around some muscle fibers (Fig. 2m-r).

**Fig. 3:**
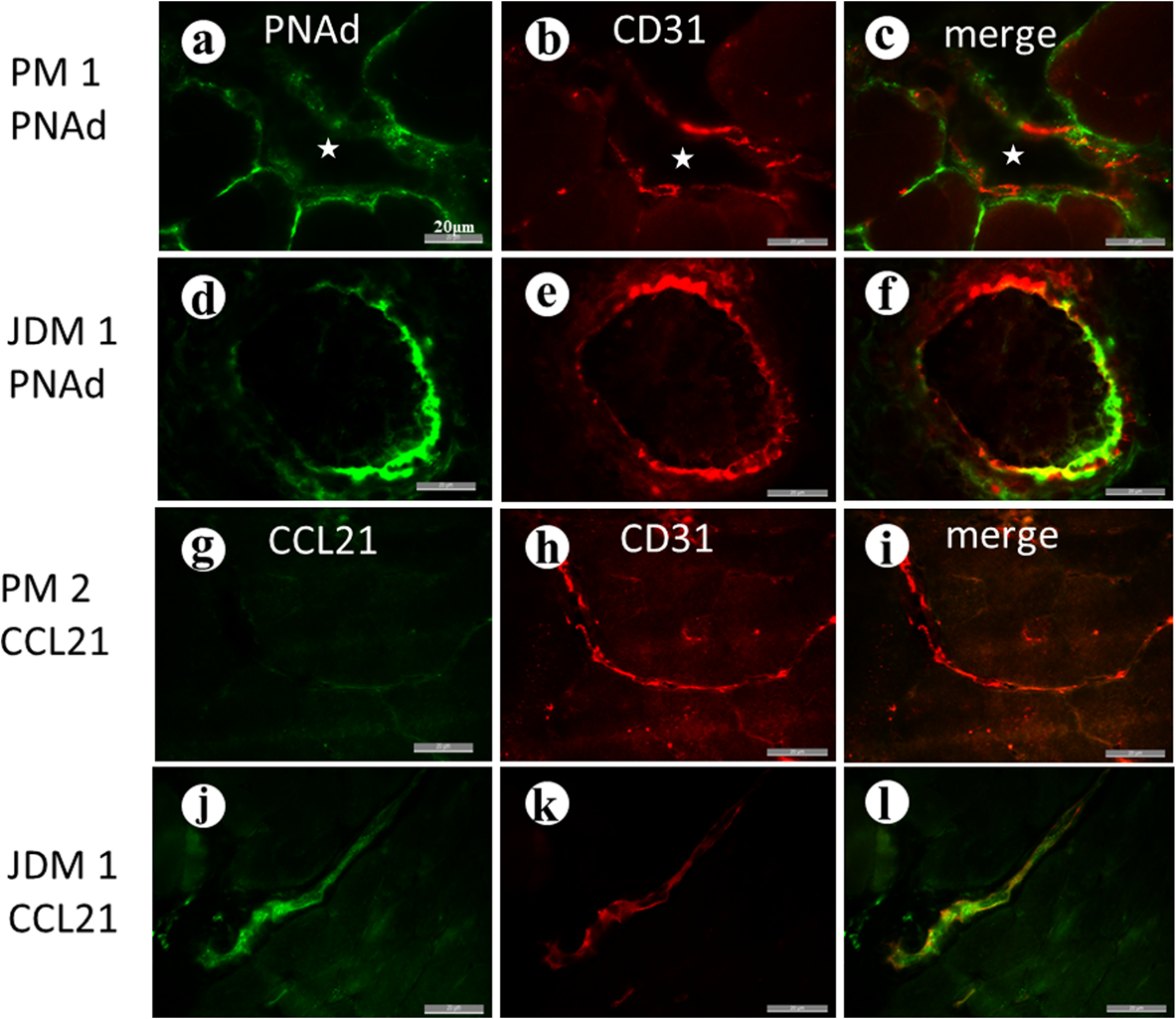
**a**-**c** In polymyositis (PM), PNAd (green) was faintly positive in a limited part of blood vessels (stars). Bars = 20 μm in all figures in Fig. 3. **d**-**f** In the case of juvenile dermatomyositis (JDM), PNAd was positive on the endothelial cells (red). **g**-**i** In PM, CCL21 was not detected. **j**-**l** Positive CCL21 in blood vessels of JDM

In two cases of PM, positivity of PNAd and CCL21 was only faintly observed in a limited stretch of blood vessels (Fig. 3a-c and g-i). In the case of JDM, both PNAd and CCL21 were detected on the limited length of endothelia cells of the blood vessels (Fig. 3d-f and j-l).

**Fig. 4:**
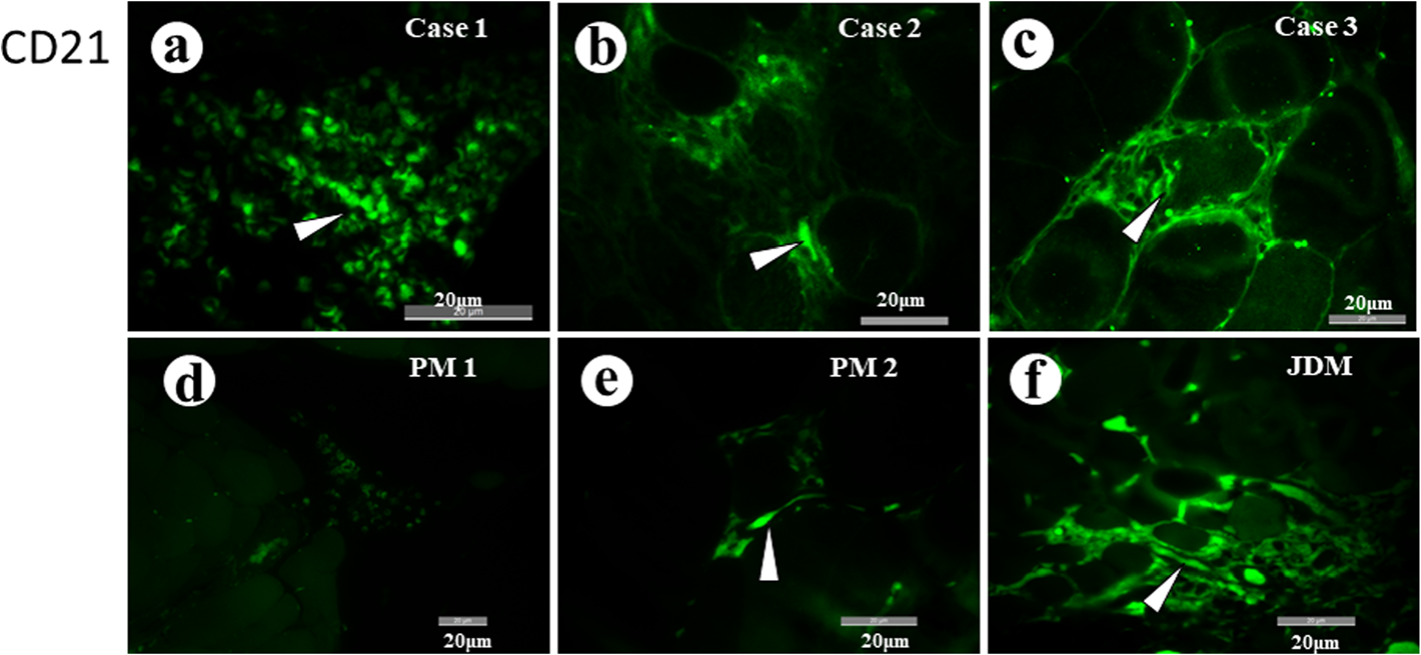
**a**-**c** Some inflammatory cells in three cases of PD1 myopathy show affinity to anti-CD21 antibody. Many of them are spindle-shaped, elongated and form ill-defined chains with occasional arborization. (arrowheads). **d** Positive cell was not seen in PM1. **e** In PM2, only a few spindle-shaped positive cells (arrowhead) were seen. **f** A case of JDM showed foci of inflammatory cells with positivity

Localization of CD21+ cells: The anti-CD21 antibody showed affinity to some inflammatory cells in three cases of PD1 myopathy. Some of the positive cells were spindle-shaped, elongated and formed clusters or ill-defined chains with arborization. They were supposed to be follicular dendritic cell (FDCs) (Fig. 4a-c). Weakly stained round cells in the background may include mature B cells. In two cases of PM, no positive cell was observed, except for a few cells around a degenerating muscle fiber in case PM2 (Fig. 4d,e). On the other hand, clusters of positive cells were seen in a case of JDM (Fig. 4f).

## Discussion

In the present study, we found almost uniform pathological features in muscle biopsies from three patients with PD-1myopathy. They were characterized by foci of inflammatory cell infiltration with degeneration of muscle fibers. The muscle fibers aberrantly expressed the MHC class I antigen. In the foci of inflammation, CD8+ cells were seen some in direct contact with the muscle fibers and others underneath the basal lamina of non-necrotic fibers as confirmed by transmission and immune-electron microscopy.

Lymphoid follicle-like structures were seen in all cases. The cells in the foci included clusters of CD21+ cells which are supposed to be FDCs [10]. Many blood vessels in the foci expressed markers of HEV, PNAd and CCL21, on the endothelial cells and in the perivascular space. Perivascular positivity of PNAd and CCL21 may be at the fibroblastic reticular cell-like cells [12]. Ultrastructural observation of the endothelial cells revealed changes consistent with those of TLO [13]. Thus, pathological features of the inflammatory foci were indicative of TLO.

TLO has been reported in allografts of skin in humans and animals [14], chronic kidney diseases [13] and variety of other forms of chronic inflammation [15]. As reviewed by Alois [16], TLO occurs in several autoimmune diseases. However, in inflammatory myopathies there have been a limited number of reports of TLO. Lopez De Padilla et al. [17] found up-regulation of CCL19 and CCL21 in the muscle of JDM, and later [18] reported TLO-like structure. It is conceivable that the presence of TLO merely reflects severe and chronic inflammation. However, cases of PM examined in the present study, which did not show TLO, had inflammation in their biopsied muscles comparable to the cases of PD-1 myopathy and had longer duration of illness than the latter.

TLO in cancer tissues has been investigated intensely since it was reported in 2008 [19]. Though its significance remains elusive, many reports have supported its favourable influence over patients’ prognosis. The immunological significance also remains controversial [20]. It enhances both humoral and cell-mediated immunity locally by producing both antibodies and effector cells, but they may not always enhance the destruction of tumor tissue, though they might suppress it.

In the present study, muscle biopsies of PM showed only faint positivity for PNAd and CCL21 in a limited stretch of the blood vessels. In PM, lymphocyte infiltration underneath the basal lamina of the muscle fibers [21] and infiltration of CD8+ T cells [22] were reported. In PD-1 myopathy we showed CD8+ cells beneath the basal lamina of muscle fibers and mononuclear cells in direct contact with the plasma membrane of the muscle fibers indicating that they are involved in the destruction of the muscle fibers. Thus CD8+ cells behave similarly in PM and PD-1 myopathy, but we saw differences between them in terms of the formation of TLO. It may explain the difference in distribution of inflammation between them. JDM showed positivity for PNAd and CCL21. Comparison between PD-1 myopathy and JDM is also puzzling. In both conditions, TLOs or TLO-like structures were observed, but infiltrating cells include more CD8+ cells in PD-1 myopathy than in JDM in which CD4+ and CD20+ cells dominate.

The naïve CD8+ cells in the tumor tissue could be activated and differentiated into effectors [23], and they can eradicate the tumor [24]. This mechanism of tissue destruction is supposed to be restricted to tumor tissues. In the present study, we examined muscle biopsies from three cases of PD-1 myopathy, two of PM, and one of JDM. Study on increased number of cases is needed before generalizing the present findings. However, observing the TLOs in non-tumorous tissue in patients who received PD-1 inhibitors, we wonder if destruction like that in tumor tissue could occur in non-tumorous tissue in a small proportion of patients treated with PD-1 inhibitor.

## Conclusions

In three cases of PD-1 myopathy, CD8+ cytotoxic T cells are likely to play a major role in damaging muscle fibers. The presence of lymphoid follicle-like structures and expression of PNAd and CCL21 on the endothelial cells suggest that TLOs are formed and may be involved in leakage of lymphocytes into the muscle tissue.

## Supplementary information

**Additional file 1: Figure S1.** “Clinical and histological features of disease control cases” in 1 page carrying text and 4 figures. **Figure S2.** “Control study: Normal sera applied in place of the primary antibodies” carrying a figure.

**Additional file 2:.** Supplementary description of methods for immunoelectron microscopy.

## Data Availability

All data and materials relevant to this article are available to referees at submission and to readers promptly upon request.

## Abbreviations

CCL21: chemokine ligand 21
CK: serum creatine kinase activity
EMG: needle electromyography
FDC: follicular dendritic cell
HEV: high endothelial venule
JDM: juvenile dermatomyositis
PD-1 myopathy: myopathy associated with PD-1 inhibitor
PD-1: programmed cell death 1
PM: polymyositis
PNAd: peripheral node addressin
TLO: tertiary lymphoid organ
