# Supplementary figures and images for: Tertiary lymphoid organs in the inflammatory myopathy associated with PD-1 inhibitors
Source: J Immunother Cancer. 2019 Sep 18;7:256. doi: 10.1186/s40425-019-0736-4 (PMC6751882; doi:10.1186/s40425-019-0736-4)

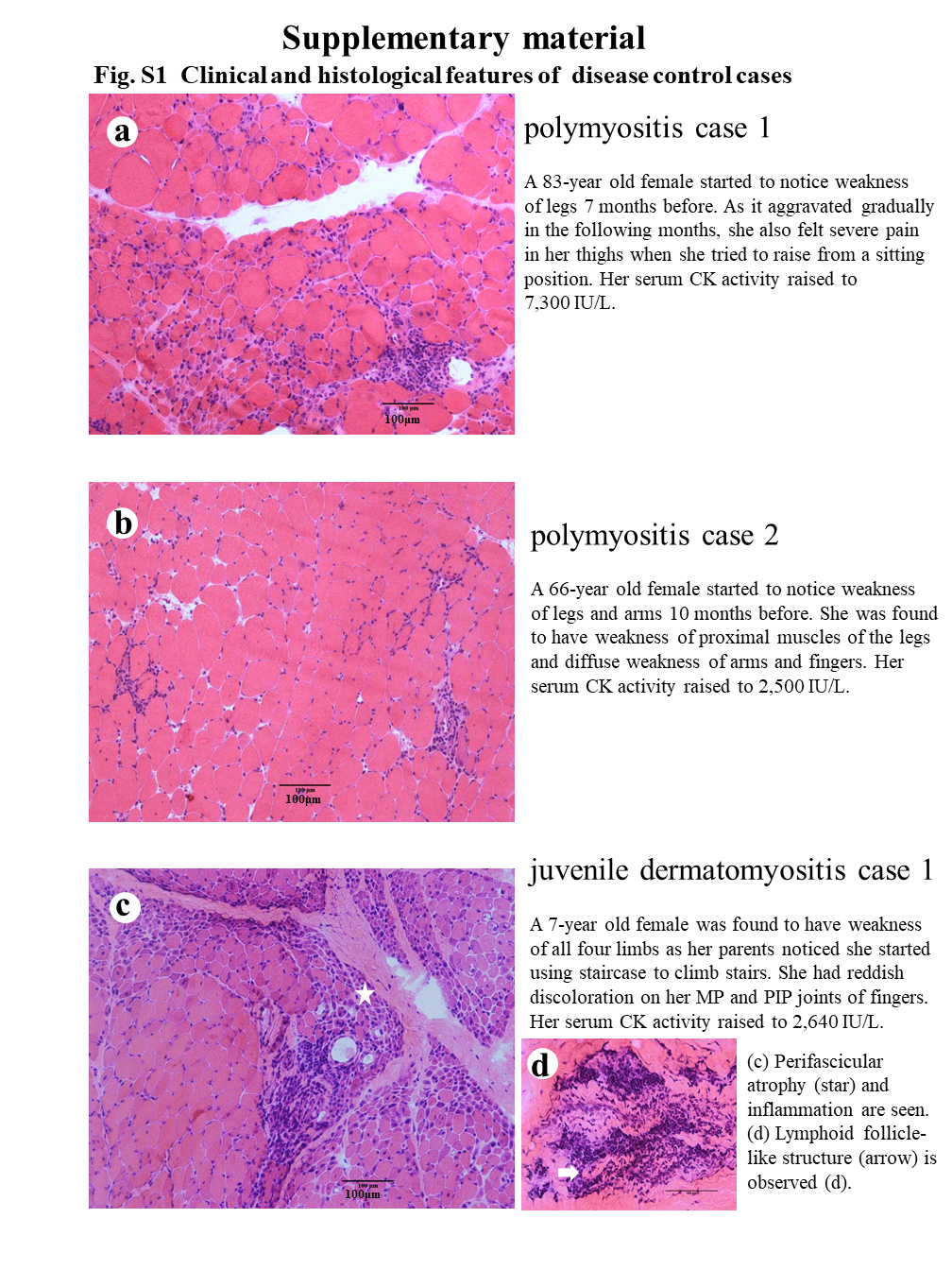

Supplement: Supplementary file 1 — Additional file 1: Figure S1. “Clinical and histological features of disease control cases” in 1 page carrying text and 4 figures. Figure S2. “Control study: Normal sera applied in place of the primary antibodies” carrying a figure. [file 40425_2019_736_MOESM1_ESM.zip › MatsubaraFigS1.TIF]

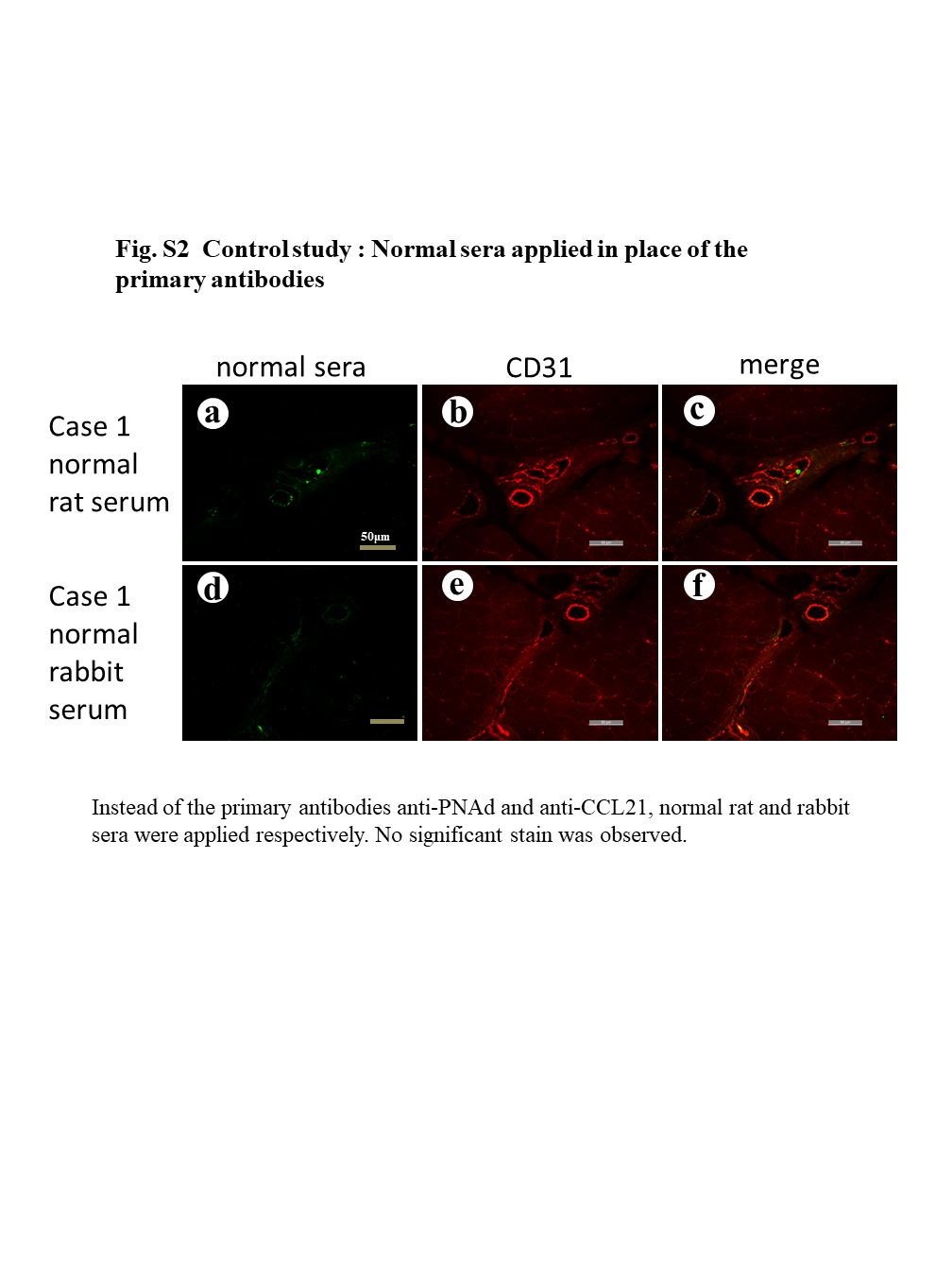

Supplement: Supplementary file 1 — Additional file 1: Figure S1. “Clinical and histological features of disease control cases” in 1 page carrying text and 4 figures. Figure S2. “Control study: Normal sera applied in place of the primary antibodies” carrying a figure. [file 40425_2019_736_MOESM1_ESM.zip › MatsubaraFigS2.TIF]
