# Supplementary material for: Tertiary lymphoid organs in the inflammatory myopathy associated with PD-1 inhibitors
Source: J Immunother Cancer. 2019 Sep 18;7:256. doi: 10.1186/s40425-019-0736-4 (PMC6751882; doi:10.1186/s40425-019-0736-4)
Supplement: Supplementary file 2 — Additional file 2:. Supplementary description of methods for immunoelectron microscopy. [file 40425_2019_736_MOESM2_ESM.docx]

## Additional supporting file 2.

Supplementary description of methods

### Immuno-electron microscopy

The frozen sections were air dried before fixation in the cold acetone followed by blocking with 2% normal rabbit serum. Anti-CD8 mouse monoclonal antibody (Dako) was applied at a dilution of ×100. After washing, anti-mouse IgG (H+L) rabbit polyclonal antibody conjugated with gold particles 25 to 30 nm in diameter (St. John’s) was applied at dilution of ×500 for 12 hours. Sections were then fixed in 1.25% glutaraldehyde for 10 minutes followed by 10 minutes of post-fixation in 2% osmium tetroxide. Then sections were dehydrated in a series of ethanol solutions from 50% to 100%, followed by propylene oxide. After application of the epoxy resin dissolved in propylene oxide, a capsule filled with the epoxy resin was placed on the sections. After polymerization, ultrathin sections, 80 nm in thickness, were cut and were stained briefly with uranyl acetate and lead citrate for 15 minutes and 1 minute, respectively.
